# A fatal yellow fever virus infection in China: description and lessons

**DOI:** 10.1038/emi.2016.89

**Published:** 2016-07-13

**Authors:** Zhihai Chen, Lin Liu, Yanning Lv, Wei Zhang, Jiandong Li, Yi Zhang, Tian Di, Shuo Zhang, Jingyuan Liu, Jie Li, Jing Qu, Wenhao Hua, Chuan Li, Peng Wang, Quanfu Zhang, Yanli Xu, Rongmeng Jiang, Qin Wang, Lijuan Chen, Shiwen Wang, Xinghuo Pang, Mifang Liang, Xuejun Ma, Xingwang Li, Quanyi Wang, Fujie Zhang, Dexin Li

**Affiliations:** 1 The National Clinical Key Department of Infectious Disease, Beijing Ditan Hospital, Capital Medical University, Beijing 100015, China; 2 National Health and Family Planning Commission of the People's Republic of China Key Laboratory for Medical Virology, Department of Viral Hemorrhagic Fever, National Institute for Viral Disease Control and Prevention, Chinese Center for Disease Control and Prevention, Beijing 102206, China; 3 Institute for Infectious Disease and Endemic Disease Control, Beijing Center for Disease Prevention and Control (CDC), Beijing 100013, China

**Keywords:** clinical investigation, genome sequence, imported case, molecular investigation, yellow fever

## Abstract

Yellow fever (YF) is a viral disease endemic to the tropical regions of Africa and South America. An outbreak of YF has been occurring in Angola, since the beginning of 2016. In March 2016, a 32-year-old Chinese man who returned from Angola was hospitalized and diagnosed with the first case of imported YF in China. Clinical observations, blood viral RNA detection, serological testing and treatments for the patient were performed daily. The virus was isolated in Vero cells, and the complete viral genome was sequenced and analyzed using the next-generation genomic sequencing platform. The patient presented with hemorrhagic fever, jaundice and oliguria at day 3 after onset, which rapidly progressed to multisystem organ failure with extremely elevated liver, pancreatic and myocardial enzymes. The patient died despite the intensive supportive treatments that were performed. A liver biopsy showed severe and multilobular necrosis. Viral RNA was detectable throughout the clinical course of the disease. Whole-genomic sequence analysis revealed that the virus belongs to the Angola71 genotype. Although the virus has been circulating in Angola for 45 years, only 14 amino-acid substitutions and no amino-acid changes were observed in the membrane and envelope proteins compared with the virus collected in 1971. The presence of this imported YF case in China indicated that with the increase in business travel among countries, YF outbreaks in Africa can lead to the international spread of the disease. The production and use of YF vaccines is, therefore, an urgent issue.

## INTRODUCTION

Yellow fever virus (YFV) is a re-emerging arbovirus of the *Flavivirus* genus, which causes an acute viral hemorrhagic disease, yellow fever (YF).^1^ The majority of the infected individuals have no symptoms or only a mild illness associated with YFV infection, but the fatality rate in severe cases can exceed 50%.^2^ Despite the fact that a safe and efficacious vaccine has been available since 1937,^3^ YF remains a public health threat in the tropical regions of Africa and South America. An outbreak of YF is currently occurring in Angola, and as of 7 April 2016, a total of 1708 cases and 238 deaths had been reported from 16 of the 18 provinces.^4^

With the rapid development of modern transportation and globalization, the virus could spread quickly to those areas with a high density of vectors and a high number of non-immune people. Imported cases had been found among travelers returned to American and European countries from Africa and South America.^5, 6, 7, 8^ However, YF has never been reported in China or in Asia. In the current study, we report the first imported fatal case of YF acquired by a Chinese man who had worked in Luanda, Angola and returned to China in March 2016. The clinical progress and viral kinetics of the case, as well as the whole-genome sequence from the first imported case are described herein.

## MATERIALS AND METHODS

### Case history

The patient was a previously healthy 32-year-old man living in China who had worked in the Luanda province, Angola since 2009. He developed a fever (39.3 °C), and chills on 8 March 2016, Beijing time, which was then defined as day 1 of disease onset. The patient returned to Beijing in the early morning of March 10 after 22 h of traveling and was admitted to Ditan Hospital on day 3 after onset. Clinical signs and symptoms were recorded, and blood samples were collected daily to monitor organ function. Serum biological parameters were assessed with automated analytical instruments. Liver tissues were analyzed after the death of the patient at day 9 for pathological analysis.

### Sample collection and laboratory tests

Serum samples were collected from the patient daily from day 3 to day 9 for RNA detection and all other clinical laboratory analyses. The YF viral RNA was detected using an in-house-designed probe and primers targeted to the NS5 gene as previously described.^9^ A standard curve with serial dilutions of known concentrations of *in vitro*-transcribed RNA (NS5 gene) from a reference plasmid was used to estimate viral load in samples. Serum samples were also tested for the presence of malaria and dengue virus, which were excluded as sources of the infection.

### Viral gene sequencing and analysis

Viral RNA was extracted from 100 μL of patient serum collected from day 3 post onset with the RNeasy Plus Mini Kit (Qiagen, Hilden, Germany, Cat# 74134) without carrier RNA. A sequencing library was constructed and analyzed with the Ion Xpress Plus Fragment Library kit (Life Technologies, Carlsbad, CA, USA), then subsequently sequenced with the Ion Torrent PGM Hi-Q OT2 kit (Life Technologies) according to the manufacturer's instructions. The next-generation genomic sequencing data were analyzed with CLC Genomics Workbench 8.5.1 (CLC bio, Aarhus, Denmark). Reads shorter than 30 bp were removed and *de novo* assembly was performed using the default parameters in the CLC software. The phylogenic analysis of E gene was performed using MEGA6 MEGA7 program software.^10, 11^ The phylogenic tree was constructed using the neighbor-joining method with the Poisson correction and a complete deletion of gaps according to the software instructions. The Atlas genome was constructed using BLAST Ring Image Generator software.^12^ The sequence was confirmed later with Vero cells and isolated YF virus from the same patient blood sample.

### Virus isolation and electronic microscopy analysis

Serum samples from the imported YF case were used for virus isolation. Serum samples were sterilized by passage through a 0.45-μM filter, and then inoculated into Vero cells. After 12 days, cell cultures were collected and passaged further into Vero cells. Supernatants were collected for identification of YF virus under electronic microscopy observation 6–7 days later. A measure of 500 μL of supernatant were ultracentrifuged at 100 000*g* for 10 min at 4 °C, and the pellet was suspended in 25 μL of phosphate-buffered saline, and absorbed on formvar and carbon-coated grids for 1 min, then stained with 1% (W/V) phosphotungstic acid (pH 6.8) for 1 min. Grids were air dried for evaluation.

### Role of the funding source

The funding source of the study had no role in the study design, data collection, data analysis, data interpretation or writing of the report. The corresponding authors had full access to all of the data in the study and had the final responsibility for the decision to submit for publication.

## RESULTS

### Clinical progress and dynamics of laboratory findings

**Figure 1:**
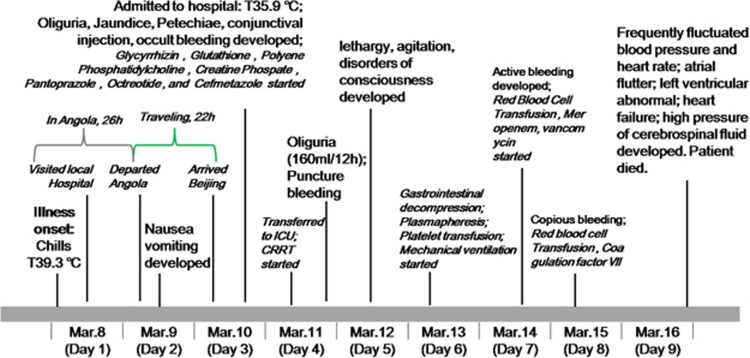
Schematic representation of the main therapeutic measures and the clinical course of the imported yellow fever case acquired in Angola.

**Figure 2:**
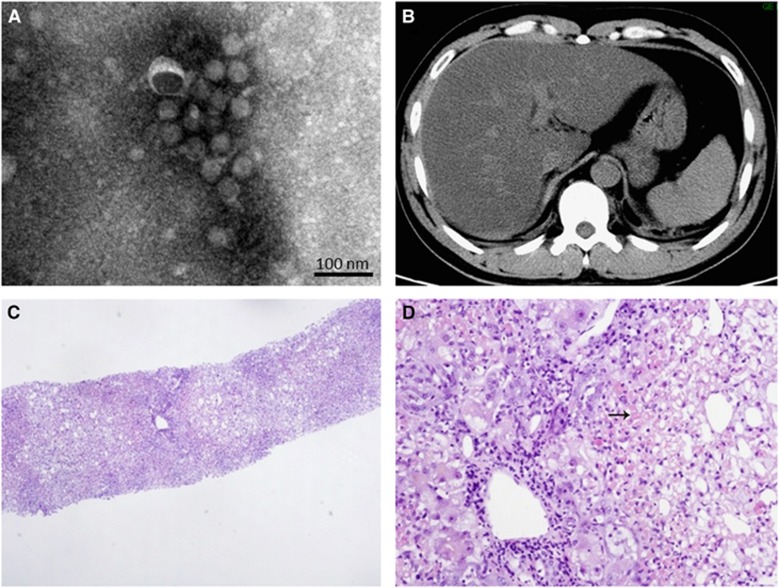
Morphology of the viral particles, as well as CT scan images and pathological characteristics of the liver. The viral particles collected in cell culture supernatants were examined with electron microscopy after negative staining (**A**). Attenuated CT value of the liver (16 HU) in a CT scan of the abdomen in contrast to the spleen (40 HU) (**B**). HE staining was performed on the liver biopsy tissue, and revealed severe multilobular necrosis (**C**, 40 ×), and panlobular and confluent hepatocytic necrosis indicated by an arrow (**D**, 200 ×). computerized tomography, CT; hematoxylin and eosin, HE.

As outlined in Figure 1, the patient developed high fever (39.3 °C) and chills on day 1 of disease onset. The patient then presented with jaundice, oliguria, gastrointestinal hemorrhage, sporadic petechiae and conjunctival congestion, but the body temperature was 35.9 °C on day 3 after admission. The patient was transferred to the intensive care unit on day 4 and underwent jugular catheterization for continuous renal replacement therapy. Fresh frozen plasma, platelet, red-cell and coagulation factor VIII supplements were administered. Advanced support treatment was provided in the form of invasive mechanical ventilation and plasmapheresis. (Figure 1; Supplementary Table S1). However, despite the maintenance of a normal temperature during disease progression, the condition did not improve. The urine volume of the patient decreased significantly. Central nervous system injury occurred on day 5; the patient presented with rapidly developing lethargy, agitation and disordered consciousness. Hepatic encephalopathy was confirmed with the presence of increased blood ammonia. In computerized tomography scan images of the abdomen, the attenuation value of the liver (16 HU) was smaller than in the spleen (40 HU; Figure 2B). Furthermore, from day 7 to day 8, the clinical condition of the patient progressively deteriorated. Epistaxis, subcutaneous hematoma, serious gastrointestinal bleeding and disseminated intravascular coagulation occurred. The patient died on day 9 after disease onset.

Laboratory values were obtained daily. YF viral RNA was detected in serum samples from the patient on day 3 after disease onset; the viral load reached 1.4 × 10^4^ copies per mL and YFV infection was confirmed. Viremia was persistent; the dynamic viral load test revealed that viral load was 1.4 × 10^4^ copies per mL on day 3 and decreased, but maintained at a level ranging from 10^3^ to 10^2^ copies per mL from day 5 to day 9, the day that the patient died (Table 1). The YF virus was isolated from the patient serum sample collected on day 3 (Figure 2A). Multiple organ failure occurred rapidly on day 3, despite the fact that the body temperature had decreased to a normal level, and the dynamic laboratory testing demonstrated significantly increased total bilirubin 100.6 μmoL/L, direct bilirubin 77.6 μmoL/L, hydrogen nitride 105 mmoL/L, lactate dehydrogenase 6276 U/L, creatine kinase 670.2 U/L, myohemoglobin 675 ng/mL, blood urea nitrogen 19.33 mmoL/L, Cr 650.1 μmoL/L, amylase 218.5 U/L and lipase 207.6 U/L, with extremely high alanine aminotransferase (11 425 U/L) and aspartate aminotransferase (21 467 U/L) levels. These markers were maintained at a high level until the last day (Table 1), indicating that the patient was suffering liver and renal failure with severe coagulation disturbances, myocardial damage and pancreatitis. Coagulation was also altered in the patient. The platelet counts persistently decreased along with prothrombin time and activated partial thromboplastin time. We also observed that CD4+ and CD8+ T cells remained at low levels (Table 1). Interleukin-6 expression was increased, reaching a peak of 856.1 on day 8, the day before the patient died. Hepatic encephalopathy was confirmed by the presence of increased blood ammonia. As a result of continuous renal replacement therapy (appendix), Cr and blood urea nitrogen continually decreased; hypocalcaemia and hypophosphatemia were improved (Table 1), but oliguria developed into anuria. Finally, a liver biopsy was carried out and revealed severe multilobular necrosis. The hepatitis was characterized by panlobular and confluent hepatocyte necrosis (Figures 2C and 2D).

### Complete viral genomic sequence analysis

**Figure 3:**
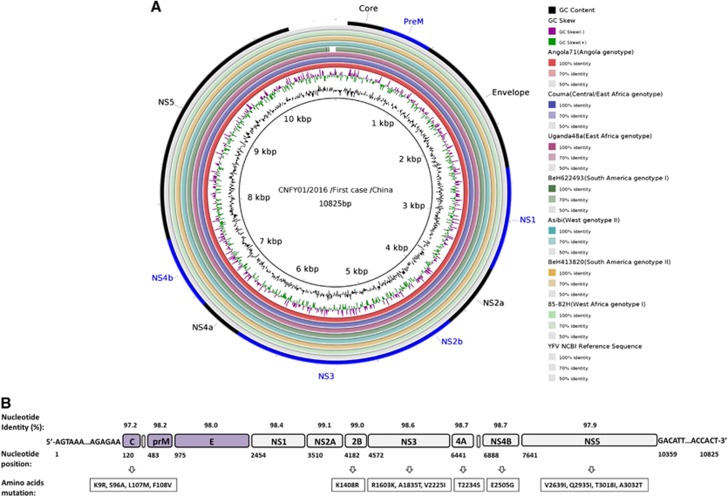
Complete genome sequence analysis of YFV strain CNYF01/2016. (**A**) A BRIG analysis of the YFV genome CNYF01/2016. The innermost (black) circle corresponds to the CNYF01/2016 complete genome of 10 823 bases in length obtained from the first imported case and used as a reference sequence (KU921608). The circles of seven colors radiating outward correspond to Angola71 (AY968064), Couma (DQ235229), Uganda48a (AY968065), BeH622493 (JF912188), Asibi (AY640589), BeH413820 (JF912181) and 85–82H (U54798). The cycle color saturation calculated by the BRIG software corresponds to the similarity of the sequences. A YFV reference sequence (NC_002031) was used for YFV coding gene annotation (out cycle). (**B**) Overview of the complete genome of the YFV strain CNYF01/2016 (GenBank accession NO KX268355). The genome consists of 10 825 nucleotides, with a 5′-UTR, a 3′-UTR, and a long open reading frame encoding for three structural proteins (capsid, premembrane/membrane and envelope) and seven non-structural proteins (NS1, NS2A, NS2B, NS3, NS4A, NS4B and NS5). The positions of the starting nucleotide of each gene, percentage of nucleotide identities and amino-acid mutations compared with the Angola71 YFV strain (GenBank accession NO AY968064) are indicated. BLAST Ring Image Generator, BRIG; untranslated region, UTR; yellow fever virus, YFV.

**Figure 4:**
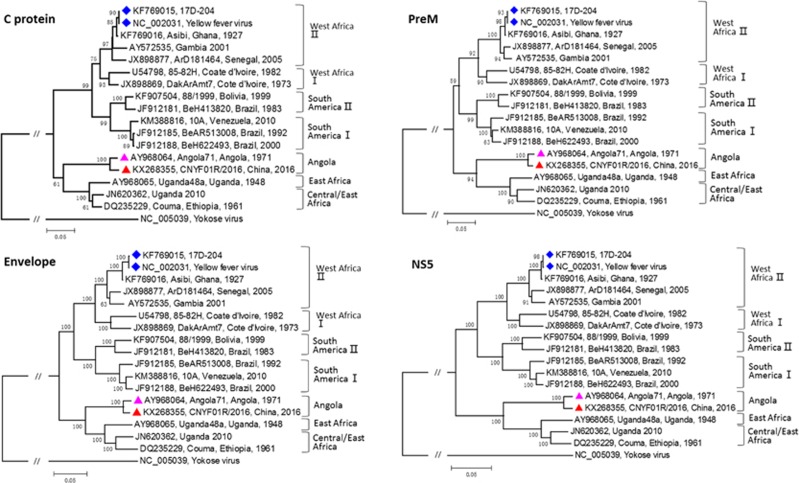
Phylogenetic analysis of C, PrM, Envelope and NS5 proteins of the Chinese YFV isolate CNYF01/2016 with 17 other selected YFV strains rooted by the Yokose virus. The trees were constructed by the neighbor-joining method based on the Jukes–Cantor model. The numbers near the nodes were bootstrap values for 1000 replicates. All YFV strains were labeled in order: accession number, strain name, country and the year of the isolation. The strain marked with a red triangle was the sequence obtained in this study, and the strain marked with a pink triangle was the only complete genome of the Angola genotype before this study. The strains marked with a blue diamond were vaccine strains. precursor to membrane, PrM; yellow fever virus, YFV.

To further confirm the clinical diagnosis and to understand the genetic information of the first imported case of YFV infection, next-generation genomic sequencing of the viral genome was performed. The complete genome sequence of strain CNYF01/2016 (GenBank NO KX268355) consisted of 10 825 bp, which was assembled from 14 428 reads and compared with other YF viral sequences from GenBank. The Atlas genome map (Figure 3A) shows the whole-genomic sequence (internal black cycle) of the CNYF01/2016. We compared the sequence to sequences from seven YFV strains from seven YFV genotypes, represented by different colors radiating outward corresponding to Angola71 (Angola genotype), Couma (Central/East Africa genotype), Uganda48a (East Africa genotype), BeH622493 (South America genotype I), Asibi (West genotype II), BeH413820 (South America genotype II) and 85–82H (West Africa genotype I). The similarities of nucleotide sequence were represented with the cycle color saturation calculated by the BLAST Ring Image Generator software; CNYF01/2016 shared 98.36%, 84.83%, 84.80%, 78.85%, 78.74%, 78.51% and 78.39% with the seven genotypes mentioned above, respectively, and 99.6%, 96.7%, 96.7%, 93.0%, 93.5%, 92.9% and 92.8% in deduced amino-acid sequences. The new isolated strain showed 98.4% identity in nucleotide sequence and 99.6% identity in amino-acid sequence. Only 14 amino-acid substitutions were detected in the YF strain CNYF01/2016; compared with strain Angola71, four substitutions were present in the C protein, one in NS2b, three in NS3, one in NS4A, one in NS4B and four in NS5, but no mutations were detected in precursor to membrane, envelope (E) or NS1 proteins (Figure 3B; Supplementary Table S2). The new isolated strain CNYF01/2016 shares 98% similarity with Angola71 in the E gene, with 29 nucleotide changes, which were all silent mutations. Phylogenetic analysis based on C, precursor to membrane, E and NS5 proteins of strain CNYF01/2016 with 17 other selected YFV strains rooted by the Yokose virus was conducted (Figure 4). The pairwise genetic distances were then calculated (Table 2). The results indicated that the CNYF01/2016 sequence was most closely related to the YFV strain Angola71 (GenBank NO AY968064), which was isolated in Angola in 1971^13^ and the complete genomic sequence was reported in 2006.^14^ Both strain CNYF01/2016 from the imported case from Angola in 2016 and strain Angola71 from 1971 shared the same Angola genotype.

## DISCUSSION

This report is the first to describe imported YF acquired by a Chinese man who had worked in the Luanda province of Angola. Despite aggressive treatment, the lethal outcome could not be prevented. The clinical features and viral kinetics of the fatal YF case were monitored and analyzed. Persistent viremia, multisystem organ failure and significant liver pathogenic damages were observed.

From the clinical data presented in this study, we postulate that multiple factors contributed to the death of the patient. First, consistent viremia may cause immunosuppression and play an important role in the progression of the disease. The viremia is usually absent during the ‘period of intoxication' of YF infection,^15^ whereas in this case, the serum viral load was persistent during the course of the disease. YFV replicates in the lymph nodes and infects dendritic cells in particular.^16^ The patient rapidly developed severe clinical symptoms, but body temperature returned to normal and stayed within normal limits until the patient's death. This finding indicates that the immune system may be damaged by the persistent viral infection. Second, the patient exhibited significant hemorrhagic symptoms, which did not improve after supplementation with platelets and plasma. It is not clear whether the damage as directly caused by the viral infection or because of a cytokine storm; this theory requires further investigation. Third, YF is a viscerotropic disease. The patient developed acute hepatic failure with a large number of necrotic liver cells detected, suggesting that advanced supportive therapy and intensive care would have made little difference in the outcome of this fulminating disease.^17^ In addition, tests for urine occult blood and urine protein were positive, indicative of renal parenchyma injury; however, renal failure was not a lethal factor. Finally, the patient had undergone 22 h of long distance travel after developing the illness, which also may have affected the disease progression.

An understanding of the pattern of divergence and dispersal of YFV in Angola was limited by the few genomic sequences published so far. Only one whole-genomic sequence of the virus circulating in Angola in 1971 was published,^13^ and the current viral strain sequence was unavailable before this study. The full-genome sequence of the YFV from this case represents the viral strain of the ongoing outbreak. Comparison of the whole genome of the current CNYF01/2016 sequence with the 1971 isolated Angola virus, only revealed 14 amino-acid changes in the whole viral genome, whereas the amino-acid sequences of the precursor to membrane, E and NS1 proteins were identical to those in Angola71. The E protein is a structural protein that plays an important function in multiple steps of the virus life cycle, including assembly, budding, attachment to target cells and viral membrane fusion, which is the major target of neutralizing antibodies.^18, 19^ NS1 is expressed on the infected cell surface or secreted into the extracellular space and antagonizes complement control of the viral infection, which would usually generate non-neutralizing, yet protective antibodies.^20^ No amino-acid changes occurred in these structural proteins, which might suggest that the antigenic characteristics and tissue tropism of the YFV circulating in Angola did not shift significantly after the outbreak in 1971. The impact of the changed nucleotides and amino acids on viral transmission and pathogenesis is still not clear; more clinical, epidemiological and genetic data are needed for further analysis.

Although our results suggest that the YFV does not substantially evolve, additional viral RNA sequencing is needed to draw any conclusions on viral evolution. A more pressing issue is global public health. The current YF outbreak in Angola exhibited a nearly 12%–50% case fatality rate.^4^ Our clinical data also indicated that YFV infection could lead to a very severe illness and death. YF has never been reported in Asia, leading to a greatly underestimated risk of YFV infection and transmission.^21, 22^ The population immunity is quite low. On the other hand, studies have shown that Asian strains of *Aedesa egypti* mosquitoes can serve as vectors for YF.^23, 24, 25^ In recent years, the scale of China–Africa trade has been rapidly increasing, which leads to an increased traveler volume between China and Africa. Therefore, the risk of YFV transmission in China is increasing, which contributes to the global risk of YF transmission. However, YF is a vaccine preventable disease. Travelers should be alerted to the risk of YF infection; vaccination against YF is of utmost importance and should be mandatory for travelers to areas, where YF is endemic to prevent the spread of the disease.

## Figures and Tables

**Table 1:** Laboratory findings in the patient

**Tests (unit)**	**Day of illness (DOI)**							**Normal range**
	**3**	**4**	**5**	**6**	**7**	**8**	**9**	
Viral load (copies per mL)	1.4 × 10^4^	3.4 × 10^3^	1.3 × 10^3^	9.3 × 10^2^	8.7 × 10^2^	4.1 × 10^2^	1.9 × 10^2^	TND
WBC (× 10^9^/L)	6.23	5.16	10.25	10.14	28.36	32.93	16.94	4–10
Neutrophils (× 10^9^/L)	5.68	4.51	8.55	8.26	21.64	29.17	14.51	2–8
RBC (× 10^12^/L)	5.19	5.82	4.44	4.43	3.65	2.98	3.24	4–5.5
Hb (g/L)	161	144	137	134	68.2	85	91	120–160
PLT (× 10^9^/L)	70	36.4	30.4	29.4	50.4	84	27.4	100–300
ALT (U/L)	11 425	10 150	5780.4	3692	1143.8	457.8	431.9	9–50
AST (U/L)	21 467	20 800	12 050	7522	2806.1	1212.4	887.6	15–40
TBIL (μmoL/L)	100.6	122.3	120.3	161.5	139.5	109.9	152.6	0–18.8
DBIL (μmoL/L)	77.6	98.8	97.3	132.8	106.2	88.2	119.8	0–6.8
NH3 (mmoL/L)	105	126	127	164	80	69	86	10–47
PT (s)	23.9	38.7	50.9	52.5	20.3	18.1	33.1	9.4–12.5
APTT (s)	43.3	52.3	61.5	65.7	63	52.7	123.6	25.1–36.5
PTA (%)	38	22	16	15	46	53	26	70–130
DD (mg/L)	34.79	29.35	27.85	28.31	19.02	27.2	70.44	0–5
FDP (μg/mL)	87.89	97.3	74.07	77.82	—	—	306.93	0–5
Fibrinogen (mg/dL)	241	202	117	71	119	104	55	200–400
LDH (U/L)	6276	1863.6	2043	2268	—	951	—	80–285
HBDH	6630	6500	4510	2785	—	909	—	74–182
CK (U/L)	670.2	844.7	704.5	1125.2	—	1187	—	38–174
CKMB (U/L)	82	75	60	62	—	66	—	<25
BNP (pg/mL)	33	—	—	—	510.2	389	1404.7	<100
MYO (ng/mL)	675	717.9	373.9	>1200	>1200	>1200	>1200	0–140.1
TnI (ng/mL)	—	0.030	0.043	0.126	0.134	2.752	9.743	0–0.028
CREA (μmoL/L)	650.1	671	457.1	343.1	286.7	266.9	189.8	59–104
BUN (mmoL/L)	19.33	23.29	12.29	6.32	4.48	4.28	3.15	1.7–8.3
AMY (U/L)	—	218.5	386	436	—	594	472	0–115
LPS (U/L)	—	207.6	588.5	507.8	—	470.2	443.8	5.6–51.3
K (mmoL/L)	4.38	4.19	3.94	5.17	4.61	4.48	5.35	3.50–5.30
Na (mmoL/L)	133.8	133.5	139.6	140.1	145.6	145.1	143.7	137–147
Cl (mmoL/L)	88.5	85.5	98.6	98.5	103	100.4	106.3	99–110
Ca (mmoL/L)	1.86	1.6	2.39	2.41	2.02	1.97	2.26	2.20–2.55
P (mmoL/L)	3.2	3.05	1.28	1.05	0.94	1.77	1.49	0.81–1.45
Lac (mmoL/L)	—	2.11	—	2.49	—	6.88	5.1	1.33–1.78
CRP (mg/L)	49.1	41.4	23.2	18.9	—	4.6	7	0–5
PCT (ng/mL)	0.54	—	—	2.46	1.6	3.76	6.3	<0.05
IL-6	—	—	182.6	68.96	87.84	856.1	—	<7
CD3+CD4+ T cells per μL	—	155	146	175	219	380	234	706–1125
CD3+CD8+ T cells per μL	—	124	122	122	127	526	363	323–836

Abbreviations: amylase, AMY; alanine aminotransferase, ALT; activated partial thromboplastin time, APTT; aspartate aminotransferase, AST; B-type natriuretic peptide, BNP; blood urea nitrogen, BUN; CD4^+^T lymphocytes, CD3^+^CD4^+^ T; CD8^+^T lymphocytes, CD3^+^CD8^+^ T; calcium, Ca; creatine kinase, CK; chlorine, Cl; the MB fraction of creatine kinase, CKMB; creatinine, CREA; c-reactive protein, CRP; D-dimer, DD; direct bilirubin, DBIL; fibrinogen degradation products, FDP; hemoglobin, Hb; Hhydroxybutyrate dehydrogenase, HBD; interleukin-6, IL-6; kalium, K; lactate dehydrogenase, LDH; lipase, LPS; myohemoglobin, MYO; sodium, Na; hydrogen nitride, NH3; phosphorus, P; procalcitonin, PCT; platelets, PLT; prothrombin time, PT; prothrombin time activity, PTA; red blood cells, RBC; total bilirubin, TBIL; testing not done, TND; troponin inhibitory, Tnl; white blood cells, WBC.

**Table 2:** Percent similarity of the amino-acid sequence of CNYF01R/2016 E protein compared with seven genotypes of YFV selected from GenBank

**Genotype**	**Genotype**	**Angola**		**West Africa II**					**West Africa I**		**East Africa**	**Central/East Africa**		**South America I**			**South America II**	
	**Strain name**	**CNYF01R/2016**	**Angola71**	**YFV**	**17D-204**	**Asibi**	**Gambia 2001**	**ArD181464**	**DakArAmt7**	**85–82H**	**Uganda48a**	**Couma**	**Uganda 2010**	**BeAR513008**	**BeH622493**	**10A**	**BeH413820**	**88–1999**
Angola	CNYF01R/2016	a	100	95.9	95.7	98.4	98.6	98.2	98.6	97.6	99.6	99.8	100	95.9	95.9	95.9	96.3	96.3
	Angola71		a	95.9	95.7	98.4	98.6	98.2	98.6	97.6	99.6	99.8	100	95.9	95.9	95.9	96.3	96.3
																		
West Africa II	YFV			a	99.8	97.6	97.4	97	97	96.3	95.9	95.7	95.9	94.3	94.3	94.3	94.5	94.7
	17D-204				a	97.4	97.2	96.8	96.8	96.1	95.7	95.5	95.7	94.1	94.1	94.1	94.3	94.5
	Asibi					a	99.8	99.4	99.4	98.8	98	98.2	98.4	96.3	96.3	96.3	96.6	96.8
	Gambia 2001						a	99.6	99.6	99	98.2	98.4	98.6	96.6	96.6	96.6	96.8	97
	ArD181464							a	99.2	98.6	97.8	98	98.2	96.3	96.3	96.3	96.3	96.6
																		
West Africa I	DakArAmt7								a	99	98.2	98.4	98.6	96.6	96.6	96.6	96.8	97
	85–82H									a	97.2	97.4	97.6	95.5	95.5	95.5	95.7	95.9
																		
East Africa	Uganda48a										a	99.4	99.6	95.5	95.5	95.5	95.9	95.9
																		
Central/East Africa	Couma											a	99.8	95.7	95.7	95.7	96.1	96.1
	Uganda 2010												a	95.9	95.9	95.9	96.3	96.3
																		
South America I	BeAR513008													a	100	100	96.8	97
	BeH622493														a	100	96.8	97
	10A															a	96.8	97
																		
South America II	BeH413820																a	99
	88–1999																	a

a no need to compare with same strain.
