# Supplementary material for: A fatal yellow fever virus infection in China: description and lessons
Source: Emerg Microbes Infect. 2016 Jul 13;5(7):e69–. doi: 10.1038/emi.2016.89 (PMC5141266; doi:10.1038/emi.2016.89)
Supplement: Supplementary Table S1 [file emi201689x1.pdf]

Supplementary Table S1 Clinical symptoms and treatments of the patient during the disease progression

**symptoms**	Day3	Day 4	Day 5	Day6	Day 7	Day 8	Day9
Body temperature (℃)	36.1	37	36.5	36.2	36.8	37.1	36.4
Heart rate (times/min)	74	76	79	60	70	101	113
Blood pressure (mmHg)	126/57	121/73	132/84	136/92	114/63	127/80	209/115
Alimentary tract hemorrhage	+	+	+	+	++	++	++
Petechiae, ecchymoses	+	++	+++	+++	+++	+++	+++
epistaxis	-	-	-	-	+	++	+
Puncture bleeding	-	+	++	++	++	++	++
Haematemesis(ml)				190	400	-	-
Urine volume (ml)	160/12h	40/12h	98	36	70	78	-
Disturbance of consciousness	-	-	+	++	+++	+++	+++
**Treatment**							
Venous transfusion (ml)	1250	1898	2987	3110	6886	5884	-
Gastrointestinal decompression (ml)				170	700	180	
Dehydrating amount of CRRT (ml)		390/12h	3320	2630	400	3850	
Output quantity (ml)	160/12h	430/12h	3418	3026	1570	4105	
CRRT		Yes	Yes	Yes	Yes	Yes	Yes
plasmapheresis				Yes	Yes	Yes	
Blood transfusion			Yes		Yes	Yes	
Platelet transfusion			Yes	Yes			
Plasma transfusion		Yes	Yes			Yes	
IPPV.				Yes	Yes	Yes	Yes
Coagulation factor Ⅶ						Yes	
Cefmetazole 1g qd	Yes	Yes	Yes	Yes	Yes	Yes	
Meropenem 1g q12h			Yes	Yes	Yes	Yes	Yes
Vancomycin 0.5g qd			Yes	Yes	Yes	Yes	Yes
