# Supplementary material for: A fatal yellow fever virus infection in China: description and lessons
Source: Emerg Microbes Infect. 2016 Jul 13;5(7):e69–. doi: 10.1038/emi.2016.89 (PMC5141266; doi:10.1038/emi.2016.89)
Supplement: Supplementary Table S2 [file emi201689x2.pdf]

Supplementary Table S2 The difference in amino acids sequence between the Yellow fever virus (CNFY01/2016) obtained in this study and the strain Angola71 collected in Angola in 1971.

Gene	Position(aa)	Difference in aa (Angogla71→CNFY01)
C	9	K→R
	96	S→A
	107	L→M
	108	F→V
NS2b	1408	K→R
NS3	1603	R→K
	1835	A→T
	2225	V→I
NS4a	2234	T→S
NS4b	2505	E→G
NS5	2639	V→I
	2935	Q→L
	3018	T→I
	3032	A→T
